# Altered lung biology of healthy never smokers following acute inhalation of E-cigarettes

**DOI:** 10.1186/s12931-018-0778-z

**Published:** 2018-05-14

**Authors:** Michelle R. Staudt, Jacqueline Salit, Robert J. Kaner, Charleen Hollmann, Ronald G. Crystal

**Affiliations:** Department of Genetic Medicine, Weill Cornell Medical College, 1300 York Avenue, Box 164, New York, NY USA

## Abstract

**Background:**

Little is known about health risks associated with electronic cigarette (EC) use although EC are rising in popularity and have been advocated as a means to quit smoking cigarettes.

**Methods:**

Ten never-smokers, without exposure history to tobacco products or EC, were assessed at baseline with questionnaire, chest X-ray, lung function, plasma levels of endothelial microparticles (EMP), and bronchoscopy to obtain small airway epithelium (SAE) and alveolar macrophages (AM). One week later, subjects inhaled 10 puffs of “Blu” brand EC, waited 30 min, then another 10 puff; *n* = 7 were randomized to EC with nicotine and *n* = 3 to EC without nicotine to assess biological responses in healthy, naive individuals.

**Results:**

Two hr. post-EC exposure, subjects were again assessed as at baseline. No significant changes in clinical parameters were observed. Biological changes were observed compared to baseline, including altered transcriptomes of SAE and AM for all subjects and elevated plasma EMP levels following inhalation of EC with nicotine.

**Conclusions:**

This study provides in vivo human data demonstrating that acute inhalation of EC aerosols dysregulates normal human lung homeostasis in a limited cohort of healthy naïve individuals. These observations have implications to new EC users, nonsmokers exposed to secondhand EC aerosols and cigarette smokers using EC to quit smoking.

**Trial registration:**

ClinicalTrials.gov NCT01776398 (registered 10/12/12), NCT02188511 (registered 7/2/14).

**Electronic supplementary material:**

The online version of this article (10.1186/s12931-018-0778-z) contains supplementary material, which is available to authorized users.

## Background

Electronic cigarettes (EC) are lithium-ion battery powered devices able to deliver aerosols often containing 4 to 24 mg nicotine per puff, as well as flavorings, propylene glycol and glycerin [1–5]. An electronic sensor detects the change in airflow upon suction and activates an element that vaporizes the liquid, flavorings and the propylene glycol and/or glycerol propellant. Many EC look like cigarettes and EC aerosols are inhaled like cigarette smoke; however, some EC devices can be individually customized to deliver aerosols at preferred settings. The use of EC is rapidly gaining popularity [1–4]. It is estimated that 4 to 6% of UK and 8 to 10% of US smokers have used EC, and there is increasing initiation of EC smoking in younger age groups with no prior smoking history [1–3, 6, 7]. EC are marketed as a substitute for cigarettes that deliver nicotine but not the toxic products of cigarette smoke [8–10], and are used as a strategy to reduce cigarette smoking, with the concept that EC are “safer” [2, 3, 8, 9, 11]. This view has been supported by reports from the Royal College of Physicians [3] and Public Health England [2] encouraging cigarette smokers to switch to EC as a strategy for cigarette smoking cessation.

While the health risks to smoking EC may or may not be less than that of smoking cigarettes, this does not mean that smoking EC is harmless to human health, particularly to the lung, the organ that receives the initial brunt of inhaled EC aerosols. EC aerosols typically contain nicotine, together with a variety of flavorings, additives and other contaminants that have the potential to affect normal lung biology [3, 5]. The human airway epithelium expresses nicotine receptors, exposure of epithelia to nicotine activates nicotine-related pathways, and in vitro studies have demonstrated that EC aerosols can modify epithelial and endothelial cell biology [12–16]. While these in vitro studies suggest that EC aerosols could be potentially harmful to the human lung, there are no studies assessing that effects of using EC on the biology of the naive human lung in vivo.

To begin to address this issue, and to circumvent the confounding effects of prior cigarette smoking, we designed a study to answer a simple question: what are the consequences to the biology of the human lung of acute exposure to healthy never smokers to EC aerosols? Our study design was limited to a small cohort of 10 healthy never smoker volunteers based on ethical concerns of exposing larger numbers of individuals to nicotine and EC without knowing the effects and potential risk to addiction. Therefore, ten volunteer healthy never smokers were fully assessed before and after acute exposure to inhalation of EC aerosols, with 7 inhaling aerosols from EC with nicotine, and 3 inhaling aerosols from identical EC without nicotine. Using fiberoptic bronchoscopy to assess the transcriptome of the small airway epithelium (SAE), the first site of lung abnormalities in cigarette smokers [17–19] and alveolar macrophages (AM), the mononuclear phagocyte defenders of the lower respiratory tract [20, 21], and flow cytometry analysis of plasma pulmonary capillary-derived endothelial microparticles [22], we assessed whether acute exposure to these aerosols modified the biology of the SAE, AM and indirectly, pulmonary capillary endothelium.

## Methods

### Study population and biologic samples

Research subjects were evaluated at the Weill Cornell Medical College Clinical Translational and Science Center and the Department of Genetic Medicine Clinical Research Facility under IRB-approved protocols (ClinicalTrials.gov Identifier: NCT01776398 and NCT02188511). Eligibility was determined following a detailed screening visit including medical history, physical exam, complete blood chemistry and coagulation studies, liver function tests, urine analysis, chest X-ray, EKG, and full pulmonary function tests. The smoking phenotype of “never-smoker” was determined by self-reported history and confirmed by absence of tobacco metabolites in the urine (urine nicotine < 2 ng/ml, urine cotinine < 5 ng/ml; see Additional file 1: Supplemental Methods for details regarding inclusion/exclusion criteria).

Upon study enrollment, ten healthy never-smokers with no history of exposure to any tobacco products or EC, were assessed on day 1 (baseline) with a questionnaire regarding symptoms to be assessed following EC use, vital signs (blood pressure, temperature, heart rate, respiratory rate), O_2_ saturation, chest X-ray, lung function, plasma EMPs and bronchoscopy with brushings to sample the small airway epithelium (SAE; 10th–12th order bronchi) and bronchoalveolar lavage to obtain alveolar macrophages (AM) at baseline. One week later, subjects were trained how to use EC then inhaled 10 puffs of “Blu” brand EC, waited 30 min, then inhaled another 10 puffs. Of the *n* = 10 total subjects, *n* = 7 were randomized to Blu EC with nicotine and *n* = 3 to Blu EC without nicotine. Immediately after the 1st and 2nd EC exposures, the questionnaires were administered and vital signs and O_2_ saturation were assessed. Within 2 h post the 2nd EC exposure, lung function, plasma EMPs and repeat bronchoscopy with brushing and lavage were obtained. Total cell counts and cell differentials of the SAE and lavage cells were quantified. RNA-sequencing was performed on mRNA from SAE and AM collected at baseline and post-EC exposure.

### Characterization of plasma endothelial microparticles

Endothelial microparticles were quantified as previously described [22]. Blood was collected and processed within 1 h to prepare platelet-rich plasma. The supernatant was further processed within 5 min to obtain platelet-poor plasma that was stained with 3 antibodies: the constitutive endothelial marker PECAM (CD31) and the constitutive platelet-specific glycoprotein Ib (CD42b). To assess the presence of relative contribution of pulmonary capillary endothelium to the elevated EMP levels, CD42b^−^CD31+ EMPs were co-stained with anti-human angiotensin converting enzyme (ACE) based on the knowledge that ACE is abundantly expressed on pulmonary capillary endothelium [22, 23]. The optimized condition for each antibody was determined by serial dilution. EMP measurements were performed twice to ensure that the measurements were reproducible. CD42b^−^CD31+ microparticle levels were normalized to isotype controls.

### Small airway epithelium and alveolar macrophages transcriptomes

SAE was collected from 10th to 12th order bronchi using flexible bronchoscopy as previously described [18]. SAE cells were dislodged from the cytology brush by flicking into 5 ml of ice-cold Bronchial Epithelium Basal Medium (BEBM, Lonza, Basel, Switzerland) and kept on ice until processed. One-fifth of the total volume was removed for cell viability and differential analysis, and the remaining sample was immediately processed and stored in TRIzol reagent (Invitrogen, Carlsbad, CA) at − 80 °C until subsequent RNA purification.

AM were recovered by bronchoalveolar lavage (BAL) [20]. The maximum volume infused per site was 150 ml, with up to two sites per individual. Recovery of the infused volume ranged from 56.2 to 65.5% (Table 1). All recovered fluid was first filtered through gauze and centrifuged at 1250 rpm for 5 min. The cell pellet was resuspended in ACK lysing buffer (5 ml, 23 °C, 5 min; Invitrogen, Carlsbad, CA) and then washed twice in 10 ml of RPMI 1640 media (Invitrogen), containing 9% fetal bovine serum (Sigma Aldrich, St. Louis MO) and 1% penicillin and streptomycin (Invitrogen). A cell count was performed using a hemocytometer with trypan blue exclusion to assess viability. The cell pellet was diluted to a concentration of 10^6^ cells/ml. Differential cell counts were carried out on a cytocentrifuged 400 μl aliquot stained with Diff-Quik. The remaining cells were plated into 6-well plastic culture dishes at 2 × 10^6^ cells/2 ml per well and incubated at 37 °C overnight in 5% CO_2_ to allow for AM purification by adherence [21]. One-fifth of the total volume was removed for cell viability and differential analysis, and the remaining sample was immediately processed and stored in TRIzol reagent (Invitrogen, Carlsbad, CA) at − 80 °C until subsequent RNA purification.

**Table 1:** Demographics of the study population and biologic samples^a^

Parameter	Total cohort of *n* = 10		Sub-cohort exposed to EC with nicotine, *n* = 7		Sub-cohort exposed to EC without nicotine, *n* = 3	
	Baseline^b^	Post-EC^c^	Baseline^b^	Post-EC^c^	Baseline^b^	Post-EC^c^
n	10		7		3	
Sex (male/female)	5/5		4/3		1/2	
Age (yr)	40.2 ± 9.7		40.4 ± 11.2		39.7 ± 6.7	
Race (B/W/H/O)^d^	7/0/3/0		6/0/1/0		1/0/2/0	
Smoking history	None		None		None	
Pulmonary function^e^						
FVC (% predicted)	110 ± 14	107 ± 13	112 ± 16	112 ± 11	105 ± 6	98.3 ± 12
FEV1 (% predicted)	110 ± 14	107 ± 15	112 ± 15	113 ± 11	103 ± 9	91 ± 8
FEV1/FVC (% observed)	81 ± 33	81 ± 4	81 ± 3	83 ± 3	81 ± 4	76 ± 4
TLC (% predicted)	92 ± 11	92 ± 11	91 ± 11	92 ± 7	94 ± 13	91 ± 21
DLCO (% predicted)	89 ± 10	86 ± 10	88 ± 10	85 ± 13	92 ± 9	87 ± 3
O_2_ saturation	99 ± 1	99 ± 1	99 ± 1	99 ± 1	99 ± 2	98 ± 1
Bronchoalveolar lavage						
% recovery	61.7%	64.8%	64.4%	65.5%	56.2%	62.9%
Total cells recovered (x10^6^)^f^	12.1 ± 7.9	10.0 ± 6.7	10.6 ± 5.3	11.0 ± 7.6	15.5 ± 13.1	6.6 ± 2.5
Epithelial cells (%)^g^	87.0 ± 9.0	1.6 ± 1.7	1.1 ± 1.0	2.0 ± 1.8	0.3 ± 0.3	0.6 ± 0.5
Macrophages (%)^g^	92.7 ± 7.4	90.0 ± 5.7	91.1 ± 8.3	90.0 ± 6.2	96.6 ± 2.7	90.1 ± 5.6
Lymphocytes (%)^g^	5.8 ± 6.5	7.8 ± 4.8	7.2 ± 7.4	7.5 ± 4.7	2.6 ± 2.1	8.5 ± 6.2
Neutrophils (%)^g^	0.5 ± 0.5	0.50 ± 0.3	0.6 ± 0.5	0.5 ± 0.2	0.3 ± 0.4	0.5 ± 0.7
Eosinophils (%)^g^	0.1 ± 0.2	0.2 ± 0.2	0.1 ± 0.2	0.1 ± 0.2	0.1 ± 0.2	0.3 ± 0.1
Small airway epithelium^h^						
Epithelial cells (%)	99.1 ± 1.0	99.3 ± 1.0	99.2 ± 1.0	99.4 ± 0.5	98.9 ± 1.2	99.0 ± 1.8
Inflammatory cells (%)	0.86 ± 1.0	0.7 ± 1.0	0.8 ± 1.0	0.6 ± 0.5	1.1 ± 1.2	1.0 ± 1.8
Ciliated cells (%)	60.1 ± 4.5	58.3 ± 7.5	58.6 ± 4.1	58.3 ± 6.8	63.7 ± 3.4	58.2 ± 10.9
Secretory cells (%)	10.5 ± 3.4	11.1 ± 2.3	9.2 ± 2.4	10.7 ± 2.0	13.4 ± 4.1	12.1 ± 3.0
Undifferentiated cells (%)	22.3 ± 4.3	21.9 ± 6.6	22.6 ± 4.7	20.2 ± 4.3	23.3 ± 3.3	26.0 ± 10.1
Basal cells (%)	6.92 ± 3.7	7.9 ± 5.9	8.7 ± 2.7	10.3 ± 5.5	2.8 ± 2.2	2.5 ± 1.3

^a^All subjects were healthy never smokers; values shown are mean ± standard deviation unless otherwise noted

^b^Baseline – assessment at baseline prior to EC exposure

^c^Post-EC – assessment 1 week after baseline, following 2 × 10 puffs of Blu brand electronic cigarette with (*n* = 7 subjects) or without (*n* = 3 subjects) nicotine

^d^Ethnicity is indicated as: Black (B), White (W), Hispanic (H), Other (O)

^e^Lung function values shown are pre-bronchodilator. FVC - forced vital capacity; FEV1 - forced expiratory volume in 1 s; DLCO - diffusion capacity for carbon monoxide; TLC - total lung capacity; all values are presented as % predicted except for FEV1/FVC presented as % observed

^f^% recovery – volume recovered / volume infused

^g^Differential cell count prior to enrichment of alveolar macrophages (see Methods for details); after enrichment, alveolar macrophages represent ≥98% of the cell population used for RNA sequencing

^h^Cell differentials of the small airway epithelium used for RNA sequencing

For both the SAE and AM, total RNA was extracted using the TRIzol method with final sample clean-up using RNeasy columns (Qiagen, Valencia, CA). RNA quantity and quality was assessed by Nanodrop ND-1000 (Thermo Scientific, Wilmington, DE) and Bioanalyzer (Agilent Technologies, Santa Clara, CA), respectively. Total RNA (0.5 μg) was submitted to the New York Genome Center for RNA-sequencing (2 × 125 bp) on the Illumina HiSeq2500 following TruSeq v2 mRNA library prep. The data are publically available in the NCBI Gene Expression Omnibus (GEO accession number: GSE85121). Illumina HiSeq paired-end reads from NYGC RNA-Seq data were processed with STAR (2.3.1z13_r470) to align reads to the GRCh37/hg19 human reference genome and RefSeq gene definitions (2014–06-02). Gene expression quantification was performed using Cufflinks (2.2) against the RefSeq gene definitions. To correct for transcript length and coverage depth, Cufflinks converts aligned reads into fragments per kilobase of exon per million fragments sequenced (FPKM) for expression using same RefSeq gene definition. For SAE expression, values of FPKM > 0.125 were included [24], for AM expression, values of FPKM > 0.08 were included.

### Statistics

The FPKM data was imported and evaluated in Partek Genomics Suite Software version 6.6 (Partek, St. Louis, MO). Fold-change was determined as least square mean of acute aerosol inhalation of EC /least square mean matched baseline samples. A *p* value < 0.05 calculated by a Student’s t-test and a fold-change > ± 1.5 were designated as the threshold. The SAE and AM RNA-seq data was also used to identify expression of nAChR in the SAE and AM. The molecular pathways associated with the significant genes impacted by acute aerosol inhalation of EC was examined using Ingenuity Pathway Analysis.

## Results

### Clinical parameters

Other than variable reports of symptoms such as feeling light-headed, dizzy, jittery, nauseated, relaxed, tense, excited or headache, there were no consistent symptoms associated with inhaling EC with or without nicotine (Additional file 1: Table S1). Likewise, there were no consistent changes in vital signs, lung function tests, O_2_ saturation, blood carboxyhemoglobin levels or urine nicotine metabolite levels, bronchoalveolar lavage cell differentials or small airway epithelium cell differentials (Table 1, Additional file 1: Table S2A and B).

### Plasma endothelial microparticles

**Fig. 1:**
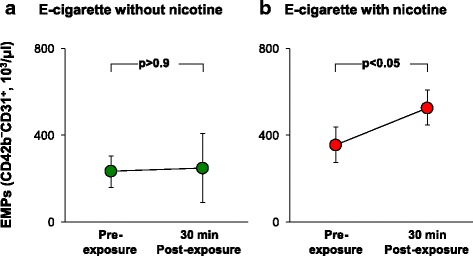
Plasma endothelial microparticle levels. Shown are data at baseline and 1 week following acute E-cigarette exposure. **a** CD42b^−^CD31+ EMP levels of never-smokers who were exposed acutely to inhalation of EC without nicotine (*n* = 3). **b** CD42b^−^CD31+ EMP levels of never-smokers who were exposed to acute inhalation of EC with nicotine (*n* = 7). For all groups before and after, the %EMP that were ACE^+^ was 76 ± 6%. Data shown are the mean ± standard error of the mean; *p* values were determined using paired, two-tailed t-test

Endothelial microparticles (EMPs) are small 0.2–1.5 μm vesicles comprised of plasma membrane and a small amount of cytosol present in circulating blood that are released from activated or injured endothelial cells [22]. Elevated levels of circulating EMP has been demonstrated in active cigarette smokers [22, 23]. To determine if EMP levels are affected following acute EC exposure, blood samples were collected at the pre-exposure baseline visit and then 1 week later at the follow up visit within 30 min following EC exposure. For both EC exposure groups, with and without nicotine, the mean %ACE^+^ CD42b^−^CD31^+^/total CD42b^−^CD31^+^ was 76 ± 6% (+nicotine vs no nicotine, *p* > 0.09), consistent with the majority of EMPs derived from pulmonary capillaries [22]. Plasma EMP levels following exposure to EC without nicotine were not significantly changed compared to baseline EMP levels (Fig. 1a). However, exposure to EC with nicotine resulted in significantly higher levels of total EMPs compared to baseline levels of total EMPs for the same individuals (Fig. 1b). These results are not unexpected, since total EMP levels are significantly higher in nicotine-containing cigarette smokers compared to nonsmoker EMP levels [22, 23], but have not yet been reported for initial EC usage.

### Expression of nicotinic acetylcholine receptor subunits

Analysis of the SAE RNA-seq data demonstrated expression of multiple nicotinic acetylcholine receptor (nAChR) subunits including α1, α3, α5, α7, α10 and β1. The AM also express nAChR subunits, including α1, α3, α5, α7, α10 and β1.

### Small airway epithelium transcriptome

**Fig. 2:**
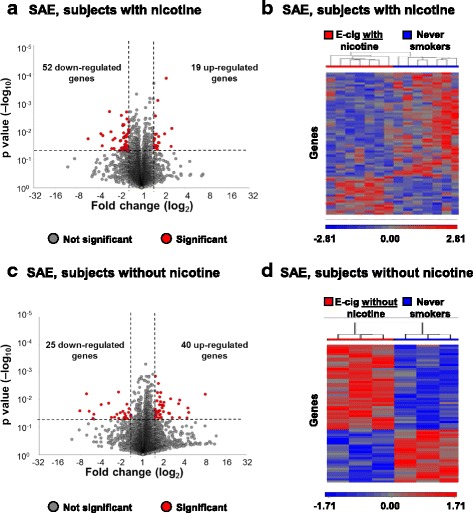
Effect of acute E-cigarette aerosol inhalation on small airway epithelium genome-wide transcriptome profiles. **a** Volcano plot showing expression of all genes comparing baseline to post-EC exposure visit from SAE of never-smokers who were exposed to acute inhalation of EC with nicotine (*n* = 7 subjects). Significance determined by *p* value < 0.05 (horizontal dashed line) and fold-change of post-EC exposure to baseline > 1.5 (vertical dashed lines). **b** Hierarchical clustering of differentially expressed genes from SAE of never-smokers who were exposed to acute inhalation of EC with nicotine (*n* = 7 subjects). **c** Volcano plot showing expression of all genes comparing baseline to post-EC exposure visit from SAE of never-smokers who were exposed to acute inhalation of EC without nicotine (*n* = 3 subjects). Significance determined by *p* value < 0.05 (horizontal dashed line) and fold-change of post-EC exposure to baseline > 1.5 (vertical dashed lines). **d** Hierarchical clustering of differentially expressed genes from SAE of never-smokers who were exposed to acute inhalation of EC without nicotine (*n* = 3 subjects)

Genome-wide gene expression profiles were assessed by mRNA-sequencing from small airway epithelium collected by brushing the 10th–12th order bronchi at baseline and again 1 wk. within 2 h of EC exposure. Using significance criteria of *p* < 0.05 and fold-change > ± 1.5, a total of 71 genes were significantly altered in SAE following exposure to EC with nicotine, including 19 up-regulated and 52 down-regulated (Fig. 2a; Additional file 1: Table S4). Acute aerosol inhalation of EC with nicotine led to global changes in SAE transcriptome profiles as observed by volcano plot (Fig. 2a) and hierarchical clustering (Fig. 2b). A total of 65 genes were significantly altered in SAE following exposure to EC without nicotine, including 40 up-regulated and 25 down-regulated (Fig. 2c; Additional file 1: Table S5). Acute aerosol inhalation of EC without nicotine also led to global changes in SAE transcriptome profiles as observed by separation of each study subject by hierarchical clustering of differentially expressed genes (Fig. 2d). Collectively in the EC users, among the pathways significantly affected was the nicotine receptor pathway (KCNK15, PPP1R16B and GNB1L) and several downstream targets of p53, including up-regulated genes (EDN1, AMOTL2, LATS2, RND3) and down-regulated genes (ATAD2, GDA, MKI67, NDC80 and RRM2), consistent with an altered activation of p53-dependent signaling.

### Alveolar macrophage transcriptome

**Fig. 3:**
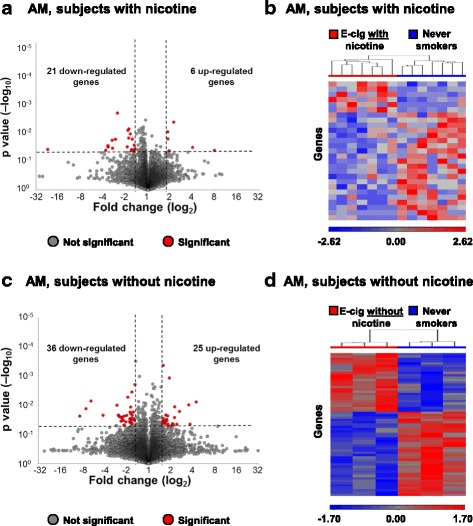
Effect of acute E-cigarette aerosol inhalation on alveolar macrophage genome-wide transcriptome profiles. **a** Volcano plot showing expression of all genes comparing baseline to post-EC exposure visit from AM of never-smokers who were exposed to acute inhalation of EC with nicotine (*n* = 7 subjects). Significance determined by *p* value < 0.05 (horizontal dashed line) and fold-change of post-EC exposure to baseline > 1.5 (vertical dashed lines). **b** Hierarchical clustering of differentially expressed genes from AM of never-smokers who were exposed to acute inhalation of EC with nicotine (*n* = 7 subjects). **c** Volcano plot showing expression of all genes comparing baseline to post-EC exposure visit from AM of never-smokers who were exposed to acute inhalation of EC without nicotine (*n* = 3 subjects). Significance determined by *p* value < 0.05 (horizontal dashed line) and fold-change of post-EC exposure to baseline > 1.5 (vertical dashed lines). **d** Hierarchical clustering of differentially expressed genes from AM of never-smokers who were exposed to acute inhalation of EC without nicotine (*n* = 3 subjects)

Genome-wide gene expression profiles were assessed by mRNA-sequencing of alveolar macrophages (AM) collected by bronchoalveolar lavage at baseline and again 1 week later within 2 h of EC exposure. Using significance criteria of *p* < 0.05 and fold-change > ± 1.5, a total of 27 genes were significantly altered in AM following exposure to EC with nicotine, including 6 up-regulated and 21 down-regulated (Fig. 3a; Additional file 1: Table S6). Acute aerosol inhalation of EC with nicotine led to global changes in AM transcriptome profiles as observed by separation of each study subject by hierarchical clustering of differentially expressed genes (Fig. 3b). A total of 61 genes were significantly altered in AM following exposure to EC without nicotine, including 25 up-regulated and 36 down-regulated (Fig. 3c; Additional file 1: Table S7). As with SAE, acute aerosol inhalation of EC without nicotine also led to global changes in AM transcriptome profiles as observed by complete separation of each study subject by hierarchical clustering (Fig. 3d). Although no dominant pathways in the AM were identified by standard pathways analysis assessing groups of up- and down-regulated genes, several genes affected by EC exposure are known to have roles in macrophage physiology and pulmonary health, including forkhead box M1 (FOXM1; FC -1.61, *p* < 1.7 × 10^− 2^, EC without nicotine, Additional file 1: Table S7), coronin 1A (CORO1A; FC -2.13, *p* < 4.7 × 10^− 2^, EC without nicotine, Additional file 1: Table S7), and prostaglandin E receptor 3 (PTGER3; FC 2.26, *p* < 2.6 × 10^− 2^, EC without nicotine, Additional file 1: Table S7).

## Discussion

The use of electronic cigarettes is increasing, with the widely held concept that EC are safer than smoking cigarettes [1–4, 6–11]. Despite the limited data on the health effects of EC on the human lung, the organ that takes the brunt of exposure to inhaled EC aerosol, the Royal College of Surgeons has recommended that “in the interest of public health, it is important to promote the use of e-cigarettes … as widely as possible as a substitute for smoking in the UK” [3]. This concept is supported by Public Health England [2]. While long term studies may eventually demonstrate that smoking EC is safer than smoking traditional cigarettes, this prompts the question: do EC aerosols have an adverse effect on the human lung? To begin to assess this question, we evaluated the biology of lung cells of healthy never smokers before and then after a brief, acute exposure to EC aerosols that is approximately equivalent in nicotine delivery to smoking 2 cigarettes. Even in this limited cohort study, we observed that acute exposure of EC aerosols to healthy naïve individuals disorders biology of at least 3 lung cell populations, including: the small airway epithelium, the initial site of cigarette smoking-induced lung abnormalities [17, 19], alveolar macrophages, the mononuclear phagocyte “defender” of the lower respiratory tract [20, 21] and, indirectly by assessment of circulating endothelial microparticles, a biomarker for health of the pulmonary capillary endothelium of the alveolar vascular bed [22, 23]. While larger studies will be required to determine if these biologic changes translates into an increased risk for lung disease, the data suggests that EC aerosols are not benign. These observations raise the concern as to whether it is premature for the medical community to proactively recommend EC use as a cigarette smoking alternative until more studies are carried out.

### Components in EC aerosols possibly relevant to lung health

EC aerosols contain nicotine and a variety of other chemicals. Nicotine is capable of evoking extensive cellular changes in cells including proliferation, cell growth and apoptosis via activation of intracellular kinase signaling pathways [16]. Nicotine displaces the local cyto-transmitter acetylcholine (Ach) from nicotinic ACh receptors (nAChRs) which are composed of 5 subunits that form hetero- or homomeric pentamer channels made of either 5 identical α subunits or combinations of α and β subunits [25]. Nine different types of α subunits (α2– α10) and 3 types of β subunits (β2– β4) have been identified. Both the human airway epithelium and AM express multiple nAChR subunits, and it is likely that the effects of nicotine exposure on the epithelium and AM occur, at a minimum, in a nAChR-dependent manner.

The finding from our study that EC use significantly altered expression of multiple genes in the nicotine receptor pathway in the small airway epithelium further supports this concept. However, nicotine exposure may have biological effects on the lung independent of nACR-mediated signaling. A recent study by Lee et al. [26] demonstrated that exposure of mice to e-cigarette smoke for 12 wk. induced DNA damage in multiple organs including the lung via the production of DNA damaging agents following nitrosation and subsequent metabolizing of nicotine. These data suggest that long-term exposure to nicotine containing e-cigarettes in humans may have similar consequences.

In addition to possible harmful effects of nicotine per se, there is a growing body of literature documenting the presence of harmful chemical constituents in EC aerosols [5]. The liquids used in EC typically contain variable ratios of vegetable glycerin, propylene glycol (PG), and nicotine and flavoring chemicals. Formaldehyde, a known degradation product of PG, reacts with PG and glycerol during vaporization to produce hemiacetals. Assessment of 42 different brands of e-liquids found formaldehyde in all 42 samples at concentrations between 0.02–10.09 mg/L [27, 28]. Other contaminants, such as limonene and various hydrocarbons [alpha-pinene, beta-pinene, gamma-terpinene, and benzene 1-methyl-4-(1-methlethyl) (para-cymene)] have been detected in some but not all e-liquids at levels higher than the recommended exposure limits [28]. Emission of aldehydes from EC has also been reported following heating and oxidation of the e-liquid main components, vegetable glycerin and propylene glycol [29]. Our data from EC users without nicotine demonstrates multiple gene expression changes in both the SAE and AM following acute EC exposure. Therefore, these data suggest that non-nicotine derived chemicals present in EC aerosols can induce molecular changes in cell populations critical to lung health which in the long term may lead to harmful effects.

### Evidence that EC aerosols modify the biology of lung cells

Consistent with the in vivo human data in the present study, there is in vitro and experimental animal evidence that EC aerosols modify lung cell biology. Exposure of cell lines from skin and lung to EC aerosols led to cytotoxic effects [12]. Exposure of human airway epithelial cells in vitro and mice in vivo to EC aerosols led to oxidative stress, low levels of inflammatory cell recruitment, delayed clearance of pathogens and other defects in host response [13, 15]. In addition, EC smoke exposure damages DNA and reduces repair activity in mouse lung and human lung cells in vitro [26]. Primary lung microvascular endothelial cells treated with e-liquid or condensed EC aerosol ± nicotine, resulted in increased endothelial permeability [14], and intra-tracheal administration of e-liquid to mice sensitized to ovalbumin aggravated allergen-induced airway inflammation and hyper-responsiveness [30].

### In vivo evidence that EC aerosols maybe harmful to the human lung

To our knowledge, there have been no prior direct assessments of lung biology following acute exposure of EC aerosols to smoking-naive humans. However, consistent with the disordered lung biology observed in the present study, there is literature demonstrating clinical abnormalities associated with acute inhalation of EC, including cough, decreased fractional exhaled nitric oxide, increases respiratory impedance and increased respiratory flow resistance [2, 3, 31]. Furthermore, a recent study by Reidel et al. [32] using quantitative proteomics to compare induced sputum samples from cigarette smokers, e-cigarette users, and nonsmokers demonstrated e-cigarette use results in a unique innate immune response in the lung with increased neutrophilic activation and altered mucin secretions. Our study demonstrates that, in naïve individuals who have no prior history of EC or traditional tobacco product usage, acute exposure to EC aerosols results in transcriptome changes in SAE and AM. Transcriptome changes in the SAE were more robust compared to AM responses, as demonstrated by more genes showing differential expression in the SAE in response to EC exposure. In the SAE, alteration of several downstream targets of p53 are consistent with activation of p53-dependent signaling following EC exposure. The p53 signaling pathway plays a central role in regulating multiple cellular functions including apoptosis, cell cycle arrest, senescence and the DNA damage response [33–35]. Furthermore, p53 activation is critical to prevent development of tobacco smoke-induced lung cancer [36–39]. Based on the knowledge that EC aerosols contains multiple toxic chemicals [5, 27–29] and that nicotine-derived metabolites/breakdown products induce DNA damage in the lung [26], we hypothesize that altered expression of p53 downstream targets in the SAE is indicative of a cellular response to environmental stress and/or DNA damage. If true, these data further strengthen the argument that EC are not benign and even acute exposure to their aerosols induces harmful effects.

Standard pathways analysis did not identify a dominant pathway in the AM transcriptome data, but several individual genes known to be involved in macrophage physiology and host defense were affected by EC exposure including forkhead box M1 (FOXM1), coronin-1A (CORO1A) and prostaglandin E receptor 3 (PTGER3) suggesting an altered immune response. FOXM1 encodes a transcriptional activator that is known to regulate expression of cell cycle related genes and has a role in controlling cell proliferation [40]. Foxm1 was recently shown to regulate pulmonary inflammatory responses to hyperoxia in neonatal rodent lungs [25]. In addition, murine studies have shown it is required for macrophage recruitment during lung inflammation and tumor formation [41]. Based on the decreased expression of FOXM1 in AM in response to EC exposure, we can hypothesize that AM from EC users may have an impaired migratory and inflammatory response. CORO1A encodes coronin-1A, a member of the WD repeat (~ 40 amino acid conserved region that may facilitate protein-protein interactions) protein family, which has been shown to inhibit autophagosome formation around *Mycobacterium tuberculosis*-containing phagosomes in rodent macrophages in culture [26]. Therefore, decreased expression of CORO1A following EC exposure may impair the phagocytic capabilities of AM. PTGER3 encodes prostaglandin E receptor 3, which is a G-protein coupled receptor that is one of four known receptors for prostaglandin E2 (PGE2) [42]. Deletion of PTGER3 was shown to improve pulmonary host defense and protect mice from death following *Streptococcus pneumoniae* infection [27], and other studies suggest that prostaglandins may play key roles in pulmonary host defense [43–45]. Therefore, increased AM expression of PTGER3 following EC use may increase the susceptibility of EC users to *Streptococcus pneumoniae* infection. In conjunction with prior studies demonstrating EC use is associated with an altered lung immune response in both humans [32] and mice [15] our study further supports this claim and suggests EC-dependent transcriptome changes in AM are a contributing factor.

### Consequences of disordered lung biology as a precursor to lung disease

The study of possible adverse effects of e-cigarette aerosols on lung health is complicated. There are many brands of e-cigarettes, with a variety of flavors and other additives in addition to nicotine [1–4]. Further, many studies to evaluate the consequences of e-cigarette aerosols are carried out in ex-cigarette smokers, where the lung has already been comprised to some degree [2, 3]. Because nicotine is addictive, it is not ethical to carry out studies exposing never smokers to long-term studies of chronic exposure to e-cigarette aerosols.

## Conclusions

The data in the present study suggests that even limited, acute exposure to EC aerosols dysregulates biology of the human lung in vivo. Whether or not chronic exposure to EC will result in lung disease is unknown and can only be evaluated by large scale, long-term studies of individuals who are not ex- or current cigarette smokers who have used only e-cigarettes, a study that would be challenging to carry out at present, as most e-cigarette users have had prior or current cigarette smoke exposure. However, the observed changes in the biology of the small airway epithelium, alveolar macrophages and (indirectly) lung capillary endothelium, may signal that EC use may not be as safe as has been assumed. Thererfore, recommending EC as less dangerous than cigarette smoking should be carefully considered until additional studies have been completed to determine which components of EC aerosol and patterns of use are responsible for the damage to airway biology.

## Additional file

Additional file 1: Supplemental Methods. Inclusion / exclusion criteria for heathy never smokers**. Table S1.** E-cigarette Effects Scale. **Table S2A.** Initiation of E-cigarette Vital Signs**. Table S2B.** Time point Differences of E-cigarette Vital Signs. **Table S3.** Urine Nicotine Metabolites Pre- and Post-Exposure to E-Cigarette Aerosols**. Table S4.** Small Airway Epithelium Differentially Expressed Genes Following Acute Inhalation of E-cigarettes with Nicotine. **Table S5.** Small Airway Epithelium Differentially Expressed Genes Following Acute Inhalation of E-cigarettes without Nicotine. **Table S6.** Alveolar Macrophage Differentially Expressed Genes Following Acute In-halation of E-cigarettes with Nicotine. **Table S7.** Alveolar Macrophage Differentially Expressed Genes Following Acute Inhalation of E-cigarettes without Nicotine. (PDF 199 kb)
