# Supplementary material for: Altered lung biology of healthy never smokers following acute inhalation of E-cigarettes
Source: Respir Res. 2018 May 14;19:78. doi: 10.1186/s12931-018-0778-z (PMC5950177; doi:10.1186/s12931-018-0778-z)
Supplement: Supplementary file 1 — Supplemental Methods. Inclusion / exclusion criteria for heathy never smokers. Table S1. E-cigarette Effects Scale. Table S2A. Initiation of E-cigarette Vital Signs. Table S2B. Time point Differences of E-cigarette Vital Signs. Table S3. Urine Nicotine Metabolites Pre- and Post-Exposure to E-Cigarette Aerosols. Table S4. Small Airway Epithelium Differentially Expressed Genes Following Acute Inhalation of E-cigarettes with Nicotine. Table S5. Small Airway Epithelium Differentially Expressed Genes Following Acute Inhalation of E-cigarettes without Nicotine. Table S6. Alveolar Macrophage Differentially Expressed Genes Following Acute In-halation of E-cigarettes with Nicotine. Table S7. Alveolar Macrophage Differentially Expressed Genes Following Acute Inhalation of E-cigarettes without Nicotine. (PDF 199 kb) [file 12931_2018_778_MOESM1_ESM.pdf]

Supplemental Methods

Inclusion / exclusion criteria for heathy never smokers

Inclusion criteria. All study subjects should be willing and able to provide informed consent for the follow up study with repeated bronchoscopies. Subjects will be male and female ≥ 21 yr of age. All subjects will be self-reported never-smokers whose urine nicotine and cotinine levels confirm undetectable levels (<2 ng/ml nicotine; <5 ng/ml cotinine) at entry into the study. Subjects must be willing to use electronic cigarettes in a brief, standardized exposure.

Inclusion criteria

- Males and females, age 21 or older
- Must be capable of providing informed consent
- Self-reported never-smokers, with current smoking status validated by the absence of nicotine metabolites in urine (nicotine <2 ng/ml and cotinine <5 ng/ml)
- Normal physical examination
- Good overall health without history of chronic lung disease, including asthma, and without recurrent or recent (within 3 months) acute pulmonary disease
- Normal routine laboratory evaluation, including general hematologic studies, general serologic/immunologic studies, general biochemical analyses, and urine analysis
- Not taking any medications relevant to lung disease or having an effect on the airway epithelium
- Negative HIV serology
- Normal chest X-ray (PA and lateral)
- Normal electrocardiogram (sinus bradycardia, premature atrial contractions are permissible)
- Females - not pregnant
- No history of allergies to medications to be used in the bronchoscopy procedure
- Willingness to participate in the study

Exclusion criteria

- Unable to meet the inclusion criteria
- Current active infection or acute illness of any kind
- Evidence of malignancy within the past 5 years
- Current alcohol or drug abuse
- Pregnancy

Initiation of E-cigarette Use “Symptom Scale” Questionnaire

Weill Cornell Initiation of E-Cigarette, Vital Signs and Questionnaire

Subject ID: ECIG - ☐☐☐ **Exposure Visit:** ☐ **Day Date of Visit:** ☐☐ - ☐☐ - ☐☐☐☐

E-Cigarette Effects Scale: First Inhalation – Assessed 30 minutes after 10 puffs.
(0= totally disagree, 1=mostly disagree, 2=more or less agree, 3=mostly agree, 4=totally agree)

1.	Feeling light-headed	☐ 0	☐ 1	☐ 2	☐ 3	☐ 4
2.	Feeling jittery	☐ 0	☐ 1	☐ 2	☐ 3	☐ 4
3.	Feeling dizzy	☐ 0	☐ 1	☐ 2	☐ 3	☐ 4
4.	Feeling relaxed	☐ 0	☐ 1	☐ 2	☐ 3	☐ 4
5.	Feeling nausea	☐ 0	☐ 1	☐ 2	☐ 3	☐ 4
6.	Feeling like you have vomit	☐ 0	☐ 1	☐ 2	☐ 3	☐ 4
7.	Feeling tense	☐ 0	☐ 1	☐ 2	☐ 3	☐ 4
8.	Feeling like you have a headache	☐ 0	☐ 1	☐ 2	☐ 3	☐ 4
9.	Feeling confused	☐ 0	☐ 1	☐ 2	☐ 3	☐ 4
10.	Feeling excited	☐ 0	☐ 1	☐ 2	☐ 3	☐ 4

E-Cigarette Effects Scale: Second Inhalation – Assessed 30 minutes after 10 more puffs.

11.	Feeling light-headed	☐ 0	☐ 1	☐ 2	☐ 3	☐ 4
12.	Feeling jittery	☐ 0	☐ 1	☐ 2	☐ 3	☐ 4
13.	Feeling dizzy	☐ 0	☐ 1	☐ 2	☐ 3	☐ 4
14.	Feeling relaxed	☐ 0	☐ 1	☐ 2	☐ 3	☐ 4
15.	Feeling nausea	☐ 0	☐ 1	☐ 2	☐ 3	☐ 4
16.	Feeling like you have vomit	☐ 0	☐ 1	☐ 2	☐ 3	☐ 4
17.	Feeling tense	☐ 0	☐ 1	☐ 2	☐ 3	☐ 4
18.	Feeling like you have a headache	☐ 0	☐ 1	☐ 2	☐ 3	☐ 4
19.	Feeling confused	☐ 0	☐ 1	☐ 2	☐ 3	☐ 4
20.	Feeling excited	☐ 0	☐ 1	☐ 2	☐ 3	☐ 4

E-Cigarette Effects Scale: Assessed 30 minutes after 10 puffs.
(0= totally disagree, 1=mostly disagree, 2=more or less agree, 3=mostly agree, 4=totally agree)

Initiation of E-cigarette Vital Signs Data Form

Weill Cornell Initiation of E-Cigarette, Vital Signs and Questionnaire

Subject ID: ECIG - ☐☐☐ **Exposure Visit:** ☐ **Day Date of Visit:** ☐☐ - ☐☐ - ☐☐☐☐

PART A: TO BE COMPLETED BY DGM STAFF

Baseline Assessment

Time Vital Signs Taken: __________

Vital Signs Pre-Exposure

Blood pressure ☐☐☐/☐☐ mm Hg

Temperature ☐☐☐.☐☐ °F or °C *(circle)*

Heart Rate ☐☐☐ per minute

Respiration ☐☐ per minute

Oxygen saturation ☐☐☐ %

1st Inhalation of e-cigarette (10 puffs)

Time Exposure Finished: __________

Time Vital Signs Taken: __________

Vital Signs Post-Exposure

Blood pressure ☐☐☐/☐☐ mm Hg

Temperature ☐☐☐.☐☐ °F or °C *(circle)*

Heart Rate ☐☐☐ per minute

Respiration ☐☐ per minute

Oxygen saturation ☐☐☐ %

***Have the subject complete: E-Cigarette Effects Scale – Assessed 30 minutes after 10 puff.**

2nd Inhalation of e-cigarette (10 puffs)

Time Exposure Finished: __________

Time Vital Signs Taken: __________

Vital Signs Post-Exposure

Blood pressure ☐☐☐/☐☐ mm Hg

Temperature ☐☐☐.☐☐ °F or °C *(circle)*

Heart Rate ☐☐☐ per minute

Respiration ☐☐ per minute

Oxygen saturation ☐☐☐ %

DGM Staff: ______________________ ________________________________ ____________

Print **Signature** **Date**

Supplemental Table I. E-cigarette Effects Scale[1]

Phenotype	Subject	First inhalation (10 puffs)										Second inhalation (10 puffs)									
		Feeling light-headed	Feeling jittery	Feeling dizzy	Feeling relaxed	Feeling nausea	Feeling like you have to vomit	Feeling tense	Feeling like you have a headache	Feeling confused	Feeling excited	Feeling light-headed	Feeling jittery	Feeling dizzy	Feeling relaxed	Feeling nausea	Feeling like you have to vomit	Feeling tense	Feeling like you have a headache	Feeling confused	Feeling excited
With nicotine	1	0	0	0	3	0	0	1	0	0	2	0	0	0	3	0	0	0	0	0	2
	2	0	0	0	3	0	0	0	0	0	0	1	0	1	1	0	0	0	0	0	0
	3	0	0	0	4	0	0	0	0	0	0	0	0	0	4	0	0	0	0	0	0
	4	0	0	0	4	0	0	0	0	0	0	0	1	0	3	0	0	0	1	0	0
	5	1	0	0	1	0	0	0	0	0	0	0	1	1	0	0	0	1	0	0	0
	6	0	0	0	4	0	0	0	0	0	0	0	0	0	0	0	0	0	0	0	0
	7	0	0	0	1	1	0	0	0	0	0	0	0	0	1	1	0	0	0	0	0
Without nicotine	8	0	0	0	3	0	0	0	0	0	0	2	0	0	3	0	0	0	1	0	0
	9	2	0	2	4	0	0	0	0	0	0	2	0	2	4	0	0	0	1	0	0
	10	0	0	0	2	0	0	0	0	0	0	0	0	0	2	0	0	0	0	0	0

[1] 0= totally disagree, 1=mostly disagree, 2=more or less agree, 3=mostly agree, 4=totally agree

Supplemental Table IIA. Initiation of E-cigarette Vital Signs

		Baseline					First Inhalation (10 puffs)					Second Inhalation (10 puffs)				
Phenotype	Subject	BP (mmHG)	Temp (C°)	Hr/ min	Resp/ min	O_2 saturation (%)	BP (mmHG)	Temp (C°)	Hr/ min	Resp/ min	O_2 saturation (%)	BP (mmHG)	Temp (C°)	Hr/ min	Resp/ min	O_2 saturation (%)
With nicotine	1	109/63	36.7	107	18	98	122/69	36.3	101	16	100	116/71	36.4	102	17	97
	2	105/58	35.4	53	14	100	102/55	35.5	54	16	100	114/58	35.3	57	16	100
	3	105/57	34.9	67	12	100	101/51	35.4	65	14	98	105/51	35.6	64	16	99
	4	114/62	36.2	67	14	99	108/64	36.1	70	10	99	107/70	35.7	61	10	99
	5	100/64	36.3	65	13	100	105/69	36.1	65	16	100	118/74	35.9	68	20	100
	6	105/67	36.1	60	14	97	108/71	35.5	66	12	97	111/73	36.3	75	14	99
	7	111/56	35.8	61	18	98	103/62	35.7	58	20	100	109/63	35.9	54	18	99
Without nicotine	8	102/62	36.2	66	14	99	105/68	36.2	66	10	99	109/64	36.4	74	18	100
	9	134/63	36.4	95	16	97	117/68	35.8	97	12	97	141/63	36.1	83	10	97
	10	102/55	36.7	87	14	100	104/57	36.8	84	12	97	108/68	36.3	80	10	100

Supplemental Table IIB. Time point Differences of E-cigarette Vital Signs

	1st Inhalation (10 puffs) - Baseline					Second Inhalation (10 puffs) – Baseline				
Phenotype	MAP[1] for BP (mmHG)	Temp (C°)	Hr/ min	Resp/ min	O_2 saturation (%)	MAP[1] for BP (mmHG)	Temp (C°)	Hr/ min	Resp/ min	O_2 saturation (%)
With nicotine[2]	1.3±4.7	-0.1±0.4	-0.1±4.0	0.1±2.7	0.3±1.4	4.6±5.1	0.0±0.4	0.1±7.8	1.1±3.6	0.1±1.1
Without nicotine[2]	1.6±3.7	-0.2±0.4	-0.3±2.5	-3.3±1.2	-1.0±1.7	5.6±4.5	-0.2±0.3	-3.7±10.4	-2.0±5.3	0.3±0.6
p value3	0.2	0.7	0.9	0.6	0.8	0.3	1	0.6	0.7	0.3

[1] Mean arterial pressure (MAP) = (2*diastolic pressure + systolic pressure)/3 used for blood pressure statistics.

[2] Data presented as average ± standard deviation.

[3] p value calculated using a 2-tailed Student's t-test with unequal variance.

Supplemental Table III. Urine Nicotine Metabolites Pre- and Post-Exposure to E-Cigarette Aerosols[1]

		Baseline				Post-e-cigarette exposure			
**Phenotype**	**Subject**	**Nicotine**	**Cotinine**	**Nornicotine**	**Anabasine**	**Nicotine**	**Cotinine**	**Nornicotine**	**Anabasine**
With nicotine	1	<2.0	<5.0	<2.0	<2.0	<2.0	<5.0	<2.0	<2.0
	2	<2.0	<5.0	<2.0	<2.0	<2.0	<5.0	<2.0	<2.0
	3	<2.0	<5.0	<2.0	<2.0	<2.0	<5.0	<2.0	<2.0
	4	<2.0	<5.0	<2.0	<2.0	<2.0	<5.0	<2.0	<2.0
	5	<2.0	<5.0	<2.0	<2.0	<2.0	<5.0	<2.0	<2.0
	6	<2.0	<5.0	<2.0	<2.0	<2.0	<5.0	<2.0	<2.0
	7	<2.0	<5.0	<2.0	<2.0	<2.0	<5.0	<2.0	<2.0
Without nicotine	8	<2.0	<5.0	<2.0	<2.0	<2.0	<5.0	<2.0	<2.0
	9	<2.0	<5.0	<2.0	<2.0	<2.0	<5.0	<2.0	<2.0
	10	<2.0	<5.0	<2.0	<2.0	<2.0	<5.0	<2.0	<2.0

[1] All urine nicotine metabolite values are normal for never smokers

Supplemental Table IV. Small Airway Epithelium Differentially Expressed Genes Following Acute Inhalation of E-cigarettes with Nicotine[1]

Gene symbol	Gene title	Fold-change[2]	p value
**Up-regulated genes**			
LINC01186	Long intergenic non-protein coding RNA 1186	2.21	$1.4x10^{-4}$
AJUBA	Ajuba LIM protein	1.69	$8.6x10^{-4}$
LATS2	large tumor suppressor kinase 2	1.56	$3.0x10^{-3}$
TMEM220-AS1	TMEM220 (transmembrane Protein 220) Antisense RNA 1	2.65	$8.2x10^{-3}$
AMOTL2	Angiomotin like 2	2.10	$1.3x10^{-2}$
SERPINB2	Serpin peptidase inhibitor, clade B (ovalbumin), member 2	1.61	$1.3x10^{-2}$
FDPSP2	Farnesyl diphosphate synthase pseudogene 2	1.56	$1.3x10^{-2}$
CST6	Cystatin E/M	1.74	$1.6x10^{-2}$
RND3	Rho family GTPase 3	1.53	$1.9x10^{-2}$
SND1-IT1	SND1 (staphylococcal nuclease and Tudor domain containing 1) intronic transcript 1 (non-protein coding)	1.50	$2.2x10^{-2}$
SMIM4	Small integral membrane protein 4	1.72	$2.6x10^{-2}$
EDN1	Endothelin 1	1.76	$2.6x10^{-2}$
C19orf60	Chromosome 19 open reading frame 60	1.51	$3.2x10^{-2}$
LIMD1-AS1	LIMD1 (LIM domains containing 1) Antisense RNA 1	1.61	$3.3x10^{-2}$
LOC101929224	Uncharacterized LOC101929224	1.68	$3.5x10^{-2}$
LOC101927604	Uncharacterized LOC101927604	2.59	$3.5x10^{-2}$
TRAPPC5	Trafficking protein particle complex 5	1.51	$4.0x10^{-2}$
OR7E12P	Olfactory receptor, family 7, subfamily E, member 12 pseudogene	1.61	$4.0x10^{-2}$
CCDC68	Coiled-coil domain containing 68	1.66	$4.5x10^{-2}$
**Down-regulated genes**			
BEST3	Bestrophin 3	-2.81	$2.1x10^{-3}$
MYL5	Myosin, light chain 5, regulatory	-1.62	$2.2x10^{-3}$
MT1X	Metallothionein 1X	-1.91	$2.9x10^{-3}$
SGK1	Serum/glucocorticoid regulated kinase 1	-1.60	$3.8x10^{-3}$
MT2A	Metallothionein 2A	-1.50	$4.9x10^{-3}$
LINC00882	Long intergenic non-protein coding RNA 882	-1.82	$6.0x10^{-3}$
NR1D1	Nuclear receptor subfamily 1 group D member 1	-1.76	$6.1x10^{-3}$
NEURL3	Neuralized E3 ubiquitin protein ligase 3	-1.83	$6.5x10^{-3}$
NR1D2	Nuclear receptor subfamily 1, group D, member 2	-1.52	$9.2x10^{-3}$
KIF14	Kinesin family member 14	-1.50	$9.3x10^{-3}$
ATAD2	ATPase family, AAA domain containing 2	-1.51	$1.1x10^{-2}$
CYP26A1	Cytochrome P450, family 26, subfamily A, polypeptide 1	-2.68	$1.1x10^{-2}$
RPL21	Ribosomal protein L21	-1.78	$1.2x10^{-2}$
FLJ20021	Uncharacterized LOC90024	-1.52	$1.2x10^{-2}$
LOC101929395	Uncharacterized LOC101929395	-3.70	$1.3x10^{-2}$
NDC80	NDC80 kinetochore complex component	-1.61	$1.3x10^{-2}$
CCL3L3	C-C motif chemokine ligand 3 Like 3	-3.48	$1.5x10^{-2}$
FAM124A	Family with sequence similarity 124A	-1.70	$1.6x10^{-2}$
PER3	Period circadian clock 3	-1.63	$1.6x10^{-2}$

Supplemental Table IV. Small Airway Epithelium Differentially Expressed Genes Following Acute Inhalation of E-cigarettes with Nicotine[1] (cont., page 2)

Gene symbol	Gene title	Fold-change[2]	p value
LYPD3	LY6/PLAUR domain containing 3	-1.62	$1.6x10^{-2}$
LOC100505683	Uncharacterized LOC100505683	-1.78	$1.7x10^{-2}$
HIF3A	Hypoxia inducible factor 3, alpha subunit	-1.76	$1.8x10^{-2}$
SMTNL1	Smoothelin-like 1	-2.35	$1.8x10^{-2}$
KRTAP5-7	Keratin associated protein 5-7	-5.59	$1.9x10^{-2}$
LINC01189	Long intergenic non-protein coding RNA 1189	-2.51	$2.1x10^{-2}$
RPS14P3	Ribosomal protein S14 pseudogene 3	-1.95	$2.2x10^{-2}$
SUSD3	Sushi domain containing 3	-1.98	$2.3x10^{-2}$
C19orf57	Chromosome 19 open reading frame 57	-1.66	$2.3x10^{-2}$
PPP1R16B	Protein phosphatase 1, regulatory subunit 16B	-1.62	$2.5x10^{-2}$
SGOL1	Shugoshin-like 1 (S. pombe)	-1.71	$3.0x10^{-2}$
RPS17	Ribosomal protein S17	-1.68	$3.0x10^{-2}$
RRM2	Ribonucleotide reductase M2	-1.58	$3.3x10^{-2}$
CRYBB2	Crystallin, beta B2	-3.54	$3.4x10^{-2}$
SLAMF6	SLAM (signaling lymphocytic activation molecule) family member 6	-1.67	$3.6x10^{-2}$
DRICH1	Aspartate-rich 1	-1.78	$3.8x10^{-2}$
HCAR3	Hydroxycarboxylic acid receptor 3	-1.64	$3.9x10^{-2}$
CXCL11	Chemokine (C-X-C motif) ligand 11	-1.92	$4.0x10^{-2}$
MKI67	Marker of proliferation Ki-67	-1.80	$4.1x10^{-2}$
C16orf95	Chromosome 16 open reading frame 95	-1.65	$4.1x10^{-2}$
RERG	RAS-like, estrogen-regulated, growth inhibitor	-1.68	$4.1x10^{-2}$
MSANTD3-TMEFF1	MSANTD3 (Myb/SANT DNA binding domain containing 3) - TMEFF1 (transmembrane protein with EGF like and two follistatin like domains 1) naturally-occurring readthrough	-1.73	$4.2x10^{-2}$
TMPRSS11D	Transmembrane protease, serine 11D	-2.60	$4.2x10^{-2}$
FUOM	Fucose mutarotase	-2.41	$4.2x10^{-2}$
IP6K3	Inositol hexakisphosphate kinase 3	-1.86	$4.3x10^{-2}$
PHKA2-AS1	PHKA2 [phosphorylase kinase, alpha 2 (liver)] antisense RNA 1	-1.63	$4.4x10^{-2}$
CCL17	Chemokine (C-C motif) ligand 17	-1.86	$4.5x10^{-2}$
ZBTB16	Zinc finger and BTB domain containing 16	-1.78	$4.6x10^{-2}$
GPRC5B	G protein-coupled receptor, class C, group 5, member B	-1.57	$4.7x10^{-2}$
GDA	Guanine deaminase	-1.75	$4.8x10^{-2}$
TMEM86A	Transmembrane protein 86A	-1.56	$4.9x10^{-2}$
CST7	Cystatin F (leukocystatin)	-2.03	$4.9x10^{-2}$
MAN1C1	Mannosidase, alpha, class 1C, member 1	-1.53	$5.0x10^{-2}$

[1] RNA sequencing analysis of the SAE from n=7 users of E-cigarettes with nicotine *vs* baseline naïve, never smoker SAE from matched individuals. Genes are listed based on p value of gene expression after exposure compared to pre-exposure of the same individuals.

[2] Fold-change = mean FPKM expression in E-cigarette with nicotine smokers/mean FPKM expression in never smokers.

Supplemental Table V. Small Airway Epithelium Differentially Expressed Genes Following Acute Inhalation of E-cigarettes without Nicotine[1]

Gene symbol	Gene title	Fold-change[2]	p value
**Up-regulated genes**			
LTB4R2	Leukotriene B4 receptor 2	1.52	$1.4x10^{-3}$
ISG20	Interferon stimulated exonuclease gene 20kDa	1.68	$2.6x10^{-3}$
GRHL3	Grainyhead-like 3 (drosophila)	1.82	$5.5x10^{-3}$
TXK	TXK tyrosine kinase	1.63	$5.5x10^{-3}$
CNFN	Cornifelin	8.04	$6.4x10^{-3}$
LOC101926888	Uncharacterized LOC101926888	1.75	$8.0x10^{-3}$
LGALS7B	Lectin, galactosidase binding soluble 7B	2.53	$9.3x10^{-3}$
ERCC6-PGBD3	ERCC6 (Excision Repair Cross-Complementation Group 6) - PGBD3 (PiggyBac transposable element derived 3) naturally-occurring readthrough	3.42	$1.0x10^{-2}$
LOC554206	Leucine carboxyl methyltransferase 1 pseudogene	1.60	$1.1x10^{-2}$
KLRF1	Killer cell lectin-like receptor subfamily F, member 1	1.81	$1.2x10^{-2}$
LAMTOR5-AS1	LAMTOR5 (late endosomal/lysosomal adaptor, MAPK and MTOR activator 5) antisense RNA 1	2.02	$1.3x10^{-2}$
BATF3	Basic leucine zipper transcription factor, ATF-like 3	3.30	$1.4x10^{-2}$
VAMP5	Vesicle-associated membrane protein 5	1.74	$1.5x10^{-2}$
SPDEF	SAM pointed domain containing ETS transcription factor	1.89	$1.6x10^{-2}$
GALNT14	Polypeptide N-acetylgalactosaminyltransferase 14	1.93	$1.6x10^{-2}$
SLC5A8	Solute carrier family 5 (sodium/monocarboxylate cotransporter), member 8	2.00	$1.7x10^{-2}$
S100P	S100 calcium binding protein P	1.90	$2.0x10^{-2}$
CEACAM5	Carcinoembryonic antigen-related cell adhesion molecule 5	4.50	$2.0x10^{-2}$
CTTNBP2	Cortactin binding protein 2	1.51	$2.2x10^{-2}$
LINC00589	Long intergenic non-protein coding RNA 589	1.76	$2.5x10^{-2}$
AQP6	Aquaporin 6, kidney specific	1.71	$2.6x10^{-2}$
BCL2L14	BCL2 (B-cell CLL/lymphoma 2)-like 14 (apoptosis facilitator)	1.57	$2.7x10^{-2}$
CIDECP	Cell death-inducing DFFA-like effector c pseudogene	1.99	$2.8x10^{-2}$
FGFBP1	Fibroblast growth factor binding protein 1	1.68	$2.8x10^{-2}$
SLC39A8	Solute carrier family 39 (zinc transporter), member 8	1.56	$2.9x10^{-2}$
NRN1L	Neuritin 1 like	4.62	$2.9x10^{-2}$
PRSS35	Protease, serine, 35	2.37	$3.1x10^{-2}$
GPR157	G protein-coupled receptor 157	1.57	$3.2x10^{-2}$
C2CD4A	C2 calcium-dependent domain containing 4A	2.44	$3.2x10^{-2}$
C2orf70	Chromosome 2 open reading frame 70	1.79	$3.5x10^{-2}$
CDRT15P1	CMT1A (Charcot-Marie-tooth neuropathy 1A) duplicated region transcript 15 pseudogene 1	1.56	$3.5x10^{-2}$
ADM2	Adrenomedullin 2	1.74	$3.6x10^{-2}$
SEMA3B	Sema domain, immunoglobulin domain (Ig), short basic domain, secreted, (semaphorin) 3B	1.55	$3.9x10^{-2}$
GPRC5D	G protein-coupled receptor, class C, group 5, member D	1.56	$4.2x10^{-2}$
DGUOK-AS1	DGUOK (deoxyguanosine kinase) antisense RNA 1	1.85	$4.5x10^{-2}$

Supplemental Table V. Small Airway Epithelium Differentially Expressed Genes Following Acute Inhalation of E-cigarettes without Nicotine[1] (cont., page 2)

Gene symbol	Gene title	Fold-change[2]	p value
ITPR1-AS1	ITPR1 (inositol 1,4,5-trisphosphate receptor type 1) antisense RNA 1 (head to head)	1.51	4.5×10^{-2}
SMC5-AS1	SMC5 (structural maintenance of chromosomes 5) antisense RNA 1 (head to head)	2.66	4.5×10^{-2}
C19orf73	Chromosome 19 open reading frame 73	3.41	4.5×10^{-2}
FAM178B	Family with sequence similarity 178, member B	1.70	4.6×10^{-2}
SYCE1	Synaptonemal complex central element protein 1	4.37	4.7×10^{-2}
**Down-regulated genes**			
DNASE1L2	Deoxyribonuclease I-like 2	-2.25	5.3×10^{-3}
LINC00892	Long intergenic non-protein coding RNA 892	-6.58	6.1×10^{-3}
TMEM220-AS1	TMEM220-AS1 (transmembrane protein 220) antisense RNA 1	-3.14	1.4×10^{-2}
LINC00882	Long intergenic non-protein coding RNA 882	-1.71	1.5×10^{-2}
TMEM171	Transmembrane protein 171	-4.02	1.5×10^{-2}
PLXDC1	Plexin domain containing 1	-1.58	2.1×10^{-2}
CALML5	Calmodulin-like 5	-8.21	2.6×10^{-2}
RAD51L3-RFFL	RAD51L3 (RAD51 paralog D) - RFFL (ring finger and FYVE-like domain containing E3 ubiquitin protein ligase) naturally-occurring readthrough	-6.02	2.7×10^{-2}
KCNK15	Potassium channel, subfamily K, member 15	-1.95	2.7×10^{-2}
DLGAP5	Discs, large (Drosophila) homolog-associated protein 5	-1.61	2.9×10^{-2}
GNB1L	Guanine nucleotide binding protein (G protein), beta polypeptide 1-like	-2.12	3.0×10^{-2}
ALG1L9P	Asparagine-linked glycosylation 1-like 9, pseudogene	-1.82	3.3×10^{-2}
LOC101927571	Uncharacterized LOC101927571	-5.14	3.4×10^{-2}
LOC101928370	Uncharacterized LOC101928370	-2.38	3.4×10^{-2}
NKAPL	NFKB (Nuclear Factor Kappa B) activating protein-like	-1.50	3.7×10^{-2}
LOC100128494	Uncharacterized LOC100128494	-1.78	4.0×10^{-2}
LOC100288637	OTU deubiquitinase 7A pseudogene	-1.50	4.0×10^{-2}
LOC101929395	Uncharacterized LOC101929395	-2.57	4.4×10^{-2}
PEG3-AS1	PEG3 (paternally expressed 3) antisense RNA 1	-2.86	4.5×10^{-2}
IFNE	Interferon, epsilon	-1.88	4.6×10^{-2}
C9orf106	Chromosome 9 open reading frame 106	-1.79	4.7×10^{-2}
SNX29P1	Sorting nexin 29 pseudogene 1	-1.87	4.8×10^{-2}
CTC-436P18.1	Uncharacterized LOC101928630	-5.31	4.9×10^{-2}
LINC01134	Long intergenic non-protein coding RNA 1134	-2.24	4.9×10^{-2}
PER3	Period circadian clock 3	-1.68	4.9×10^{-2}

[1] RNA sequencing analysis of the SAE from n=7 users of E-cigarettes without nicotine *vs* baseline naïve, never smoker SAE from matched individuals. Genes are listed based on p value of gene expression after exposure compared to pre-exposure of the same individuals.

[2] Fold-change = mean FPKM expression in E-cigarette with nicotine smokers/mean FPKM expression in never smokers.

Supplemental Table VI. Alveolar Macrophage Differentially Expressed Genes Following Acute Inhalation of E-cigarettes with Nicotine[1]

Gene symbol	Gene title	Fold-change[2]	p value
**Up-regulated genes**			
LINC00571	Long intergenic non-protein coding RNA 571	2.22	$4.9x10^{-3}$
RPL13P5	Ribosomal protein L13 pseudogene 5	1.84	$1.1x10^{-2}$
LOC100506368	Uncharacterized LOC100506368	1.86	$1.8x10^{-2}$
LOC100289473	Cytoskeleton associated protein 2-like pseudogene	3.99	$3.6x10^{-2}$
LAMTOR5-AS1	LAMTOR5 (late endosomal/lysosomal adaptor, MAPK And MTOR activator 5) antisense RNA 1	7.84	$4.5x10^{-2}$
ICAM4	Intercellular adhesion molecule 4 (landsteiner-wiener blood group)	1.93	$4.6x10^{-2}$
**Down-regulated genes**			
LOC101927124	Uncharacterized LOC101927124	-2.57	$2.4x10^{-3}$
AGAP2-AS1	AGAP2 (ArfGAP with GTPase domain, ankyrin Repeat and PH domain 2) antisense RNA 1	-1.81	$8.4x10^{-3}$
LOC100379224	Uncharacterized LOC100379224	-1.86	$9.4x10^{-3}$
CCNT2-AS1	CCNT2 (Cyclin T2) antisense RNA 1	-1.73	$1.2x10^{-2}$
CCDC17	Coiled-coil domain containing 17	-1.82	$1.7x10^{-2}$
LOC100507634	Uncharacterized LOC100507634	-1.62	$1.9x10^{-2}$
PANDAR	Promoter Of CDKN1A (cyclin-dependent kinase inhibitor 1A) antisense DNA damage activated RNA	-2.73	$1.9x10^{-2}$
LOC727751	Golgin A2 Pseudogene	-3.02	$2.0x10^{-2}$
P4HA2-AS1	P4HA2 (prolyl 4-hydroxylase subunit alpha 2) antisense RNA 1	-1.53	$3.0x10^{-2}$
CRIP2	Cysteine-rich protein 2	-3.45	$3.1x10^{-2}$
RPS14P3	Ribosomal protein S14 pseudogene 3	-1.90	$3.4x10^{-2}$
CRYAB	Crystallin, alpha B	-3.42	$3.4x10^{-2}$
CCL28	Chemokine (C-C motif) ligand 28	-3.45	$3.5x10^{-2}$
MTVR2	Mouse mammary tumor virus receptor homolog 2	-2.99	$3.8x10^{-2}$
PFN2	Profilin 2	-1.51	$4.1x10^{-2}$
ZASP	ZO-2 associated speckle protein	-22.28	$4.2x10^{-2}$
DNALI1	Dynein, axonemal, light intermediate chain 1	-1.66	$4.3x10^{-2}$
SNCAIP	Synuclein, alpha interacting protein	-1.57	$4.5x10^{-2}$
SUGT1P1	SUGT1 (SGT1 homolog, MIS12 kinetochore complex assembly cochaperone) pseudogene 1	-3.68	$4.5x10^{-2}$
FAM228A	Family with sequence similarity 228, member A	-1.67	$4.6x10^{-2}$
TRIM61	Tripartite motif containing 61	-1.62	$4.6x10^{-2}$

[1] RNA sequencing analysis of the Alveolar Macrophages (AM) from n=7 users of E-cigarettes with nicotine *vs* baseline naïve, never smoker AM from matched individuals. Genes are listed based on p value of gene expression after exposure compared to pre-exposure of the same individuals.

[2] Fold-change = mean FPKM expression in E-cigarette with nicotine smokers/mean FPKM expression in never smokers.

Supplemental Table VII. Alveolar Macrophage Differentially Expressed Genes Following Acute Inhalation of E-cigarettes without Nicotine[1]

Gene symbol	Gene title	Fold-change[2]	p value
**Up-regulated genes**			
C1orf159	Chromosome 1 open reading frame 159	1.59	$4.3x10^{-4}$
INTS6-AS1	INTS6 antisense RNA 1	1.92	$1.2x10^{-3}$
ULBP3	UL16 binding protein 3	4.46	$7.7x10^{-3}$
LOC284632	Uncharacterized LOC284632	3.53	$9.7x10^{-3}$
SLX1A-SULT1A3	SLX1A (SLX1 homolog A, structure-specific endonuclease subunit) - SULT1A3 (sulfotransferase family 1A member 3) naturally-occurring readthrough (NMD candidate)	2.27	$1.1x10^{-2}$
PHKG1	Phosphorylase kinase, gamma 1 (muscle)	2.65	$1.6 x10^{-2}$
ALKBH6	AlkB, alkylation repair homolog 6 (*E. coli*)	1.72	$2.2 x10^{-2}$
ZNF433	Zinc finger protein 433	1.82	$2.3 x10^{-2}$
PTGER3	Prostaglandin E receptor 3 (subtype EP3)	2.26	$2.6 x10^{-2}$
GAD1	Glutamate decarboxylase 1 (brain, 67kDa)	1.59	$2.9 x10^{-2}$
FAHD2CP	Fumarylacetoacetate hydrolase domain containing 2C, pseudogene	1.77	$3.1 x10^{-2}$
RPL13P5	Ribosomal protein L13 pseudogene 5	1.75	$3.3x10^{-2}$
SLC43A2	Solute carrier family 43 (amino acid system L transporter), member 2	1.77	$3.4x10^{-2}$
TUBA1C	Tubulin, alpha 1c	1.67	$3.5x10^{-2}$
RPP21	Ribonuclease P/MRP subunit P21	1.57	$3.6x10^{-2}$
TUBB6	Tubulin, beta 6 class V	1.69	$3.8x10^{-2}$
SMAD6	SMAD family member 6	1.68	$4.2x10^{-2}$
IGSF22	Immunoglobulin superfamily, member 22	2.12	$4.3x10^{-2}$
CCNB2	Cyclin B2	1.54	$4.4x10^{-2}$
TMEM88	Transmembrane protein 88	3.68	$4.5x10^{-2}$
GPR68	G protein-coupled receptor 68	2.44	$4.5x10^{-2}$
PROCR	Protein C receptor, endothelial	1.84	$4.5x10^{-2}$
LOC101928865	Uncharacterized LOC101928865	2.18	$4.6x10^{-2}$
HIST1H1C	Histone cluster 1, H1c	2.06	$5.0x10^{-2}$
C21orf140	Chromosome 21 open reading frame 140	1.83	$5.0x10^{-2}$
**Down-regulated genes**			
APOBEC3F	Apolipoprotein B mRNA editing enzyme, catalytic polypeptide-like 3F	-1.53	$1.9x10^{-3}$
PSMG4	Proteasome (prosome, macropain) assembly chaperone 4	-1.69	$3.5x10^{-3}$
LYPD5	LY6/PLAUR domain containing 5	-1.92	$5.3x10^{-3}$
SETSIP	SET-like protein	-6.08	$7.1x10^{-3}$
LINC00957	Long intergenic non-protein coding RNA 957	-2.70	$9.9x10^{-3}$
SH3BGR	SH3 domain binding glutamate-rich protein	-1.76	$1.1x10^{-2}$
KRTCAP3	Keratinocyte associated protein 3	-7.13	$1.3x10^{-2}$
FOXM1	Forkhead box M1	-1.61	$1.7x10^{-2}$

Supplemental Table VII. Alveolar Macrophage Differentially Expressed Genes Following Acute Inhalation of E-cigarettes without Nicotine[1] (cont., page 5)

Gene symbol	Gene title	Fold-change[2]	p value
CDK6	Cyclin-dependent kinase 6	-1.79	$1.7x10^{-2}$
PMS2P4	Postmeiotic segregation increased 2 pseudogene 4	-1.75	$1.9x10^{-2}$
CHST10	Carbohydrate sulfotransferase 10	-2.58	$2.2x10^{-2}$
VNN3	Vanin 3	-1.67	$2.2x10^{-2}$
SIT1	Signaling threshold regulating transmembrane adaptor 1	-4.17	$2.2x10^{-2}$
FAM86JP	Family with sequence similarity 86 member J, pseudogene	-1.99	$2.2x10^{-2}$
CMTM2	CKLF (chemokine-like factor)-like MARVEL transmembrane domain containing 2	-8.66	$2.4x10^{-2}$
TRAF3IP3	TRAF3 (TNF receptor associated Factor 3) interacting protein 3	-2.53	$2.4x10^{-2}$
GCSAM	Germinal center-associated, signaling and motility	-2.16	$2.4x10^{-2}$
CD244	CD244 molecule, natural killer cell receptor 2B4	-2.51	$2.4x10^{-2}$
FBF1	Fas (TNFRSF6) binding factor 1	-2.37	$2.6x10^{-2}$
TBX6	T-box 6	-2.28	$2.6x10^{-2}$
RAVER2	Ribonucleoprotein, PTB-binding 2	-2.30	$2.8x10^{-2}$
SPATA18	Spermatogenesis associated 18	-1.72	$2.8x10^{-2}$
C1orf213	Chromosome 1 open reading frame 213	-1.58	$2.9x10^{-2}$
ANKRD29	Ankyrin repeat domain 29	-1.81	$2.9x10^{-2}$
WDR17	WD repeat domain 17	-1.69	$3.0x10^{-2}$
FAM84B	Family with sequence similarity 84, member B	-2.22	$3.3x10^{-2}$
YIF1B	Yip1 interacting factor homolog B (S. cerevisiae)	-1.56	$3.3x10^{-2}$
CEACAM1	Carcinoembryonic antigen-related cell adhesion molecule 1 (biliary glycoprotein)	-2.38	$3.5x10^{-2}$
LOC153684	Uncharacterized LOC153684	-1.74	$3.6x10^{-2}$
FGD1	FYVE, RhoGEF and PH domain containing 1	-2.04	$3.7x10^{-2}$
TLN2	Talin 2	-1.82	$3.7x10^{-2}$
ITGA6	Integrin, alpha 6	-1.71	$4.0x10^{-2}$
HLA-DOA	Major histocompatibility complex, class II, DO alpha	-1.75	$4.0x10^{-2}$
ANKMY1	Ankyrin repeat and MYND domain containing 1	-1.68	$4.4x10^{-2}$
CORO1A	Coronin, actin binding protein, 1A	-2.13	$4.7x10^{-2}$
TTC28-AS1	TTC28 (tetratricopeptide repeat domain 28) antisense RNA 1	-2.17	$4.9x10^{-2}$

[1] RNA sequencing analysis of Alveolar Macrophages (AM) from n=7 users of E-cigarettes without nicotine *vs* baseline naïve, never smoker AM from matched individuals. Genes are listed based on p value of gene expression after exposure compared to pre-exposure of the same individuals.

[2] Fold-change = mean FPKM expression in E-cigarette with nicotine smokers/mean FPKM expression in never smokers.
